# A case of tuberculous peritonitis

**DOI:** 10.3892/etm.2012.717

**Published:** 2012-09-20

**Authors:** WENYI NIU, MINGYING LI, YONGGAN WAN, HELIN WANG, ZHENXUAN WU, JUNWEI CUI

**Affiliations:** Department of Tuberculosis, The First Affiliated Hospital of Xinxiang Medical College, Weihui 453100, P.R. China

**Keywords:** *Mycobacterium tuberculosis*, peritoneal adhesion, ascites, laparoscope

## Abstract

This study aimed to provide the initial laparoscopy results as well as the results after a 16-month follow-up of a 78-year-old patient with tuberculous peritonitis. Imaging and laboratory examinations were performed for preliminary diagnosis, and laparoscopy and Gram staining were used for definitive diagnosis. The initial laparoscopy results showed the presence of typical yellow-white nodules on the liver surface and a biopsy demonstrated caseous necrotic granuloma. After 16 months, laparoscopy results showed that yellow-white nodules were reduced after antituberculous drug treatment and adhesions formed by fibrin networks were clearly visible. Laparoscopy and biopsy contributed to the rapid diagnosis of tuberculous peritonitis.

## Introduction

Tuberculosis is an infectious disease, but its incidence rate has exhibited a gradual decrease (1). There are often reports on laparoscopy results of abdominal tuberculosis peritonitis. When a patient presents with unexplained ascites, laparoscopy may contribute to the diagnosis of this disease (2). In this study, we describe the apparent changes in the laparoscopy results from a female with tuberculous peritonitis during the 16-month illness.

## Materials and methods

### General data

**Figure 1:**
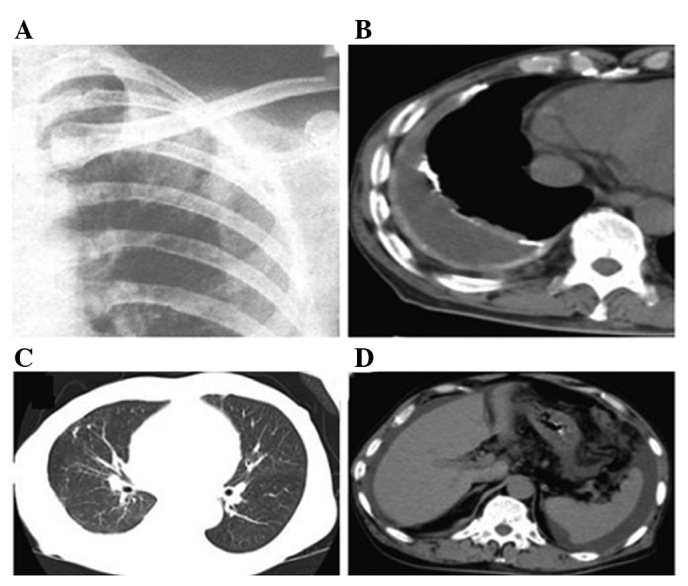
Imaging examination results of the patient with tuberculous peritonitis: (A and B) thoracic hydrops, (C) calcification foci caused by previous thoracic pulmonary tuberculosis and (D) ascites around the liver and spleen.

On January 12, 2010, a 78-year-old female with a fever of 39°C and abdominal distention symptoms was transferred from the Emergency Department to the Department of Tuberculosis, The First Affiliated Hospital of Xinxiang Medical College (Weihui, China). Upon hospitalization, physical examination results of the patient were as follows: body temperature, 38°C; blood pressure, 163/73 mmHg; heart rate, 89 beats/min; and stable vital signs. The following results indicated the presence of inflammation in the body: leukocyte count, 34×10^9^/*μ*l; rod nuclear cells, 85.5%; erythrocyte sedimentation rate (ESR), 28 mm/h; C-reactive protein (CRP), 75 mg/l; yet normal liver and kindey functions. In addition, the tuberculin test diameter was 10×9 mm. However, chest X ray and pectoral and abdominal CT demonstrated that there was hydrops in the chest and abdomen. As the patient had previously suffered from pulmonary tuberculosis at 27 years of age, the lung presented with calcification foci caused by previous pulmonary tuberculosis (Fig. 1). Fever, CT examination results and inflammation symptoms suggested that the patient suffered from tuberculosis.

### Laboratory examination

The Rivalta test result of the ascites was positive and the adenosine deaminase (ADA) level was high (117.8 U/l). However, the conventional staining and culture test results of *Mycobacterium tuberculosis* in the ascites were negative, and bacteria or tumor cells were undetectable.

### Treatment

Continuous antituberculous drugs isoniazid (INH; 0.4 g/day), rifampin (RFP; 0.45 g/day), pyrazinamide (PZA; 1.5 g/day) and ethambutol (EB; 1 g/day), and antibiotics imipenem/cilastatin (IPM/CS) were administered for treatment. The following day, the fever disappeared. After IPM/CS administration was stopped, the body temperature of the patient increased. Therefore, antibiotic treatment was conducted continuously and ascites gradually reduced. After one month, the febrile symptom recurred, and EB administration was stopped and replaced with streptomycin (SM) treatment (1 g, 3 days/week). Subsequently, the body temperature was restored to the normal level, and systemic conditions were improved and became gradually stable.

## Results

### Laparoscopy

**Figure 2:**
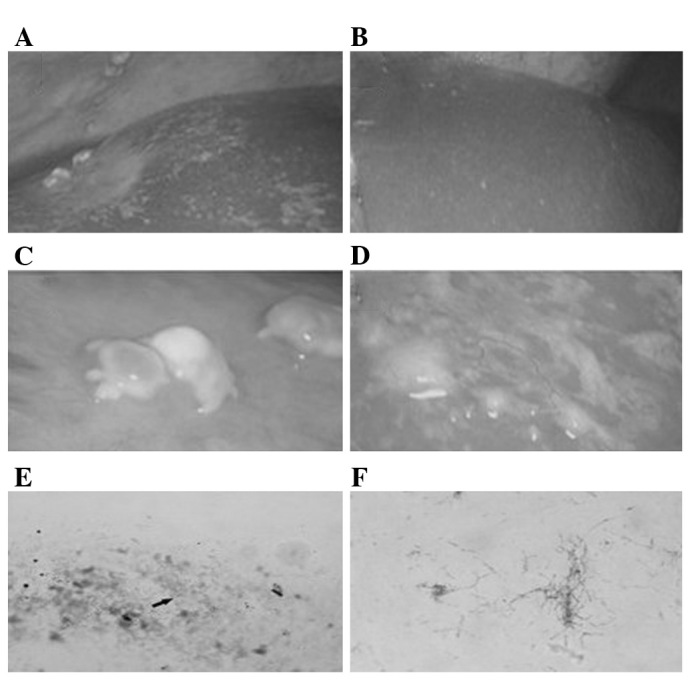
Laparoscopy results. The liver capsule became white in the (A) right and (B) left lobes of the liver. (C) The left lobe of the liver also had typical yellow-white nodules (D) and numerous white small nodules. (E and F) *Mycobacterium tuberculosis* Gram staining result of nodules selected under the laparoscope was positive.

**Figure 3:**
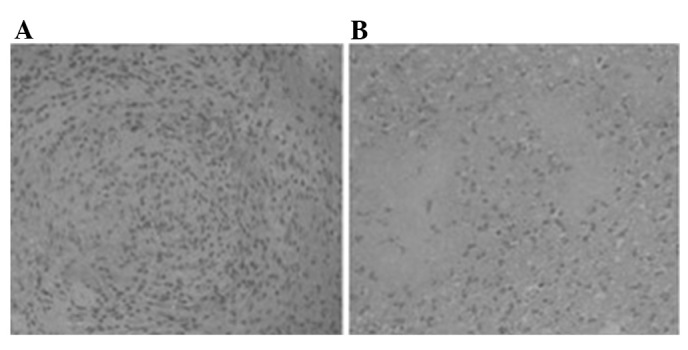
Liver biopsy results under laparoscope. (A) Epithelioid cell granuloma and (B) caseous necrosis.

As shown in Fig. 2, it was clear that the liver capsule became white in the right and left lobes of the liver (Fig. 2A and B, respectively). The left lobe of the liver also had typical yellow-white nodules (Fig. 2C) and numerous white small nodules (Fig. 2D). Fig. 2E and F shows that the *Mycobacterium tuberculosis* Gram staining result of nodules selected under the laparoscope was positive. Fig. 3A and B shows epithelioid cell granuloma and caseous necrosis, repectively. These results were in agreement with the diagnosis of tuberculous peritonitis.

### Second laparoscopy

**Figure 4:**
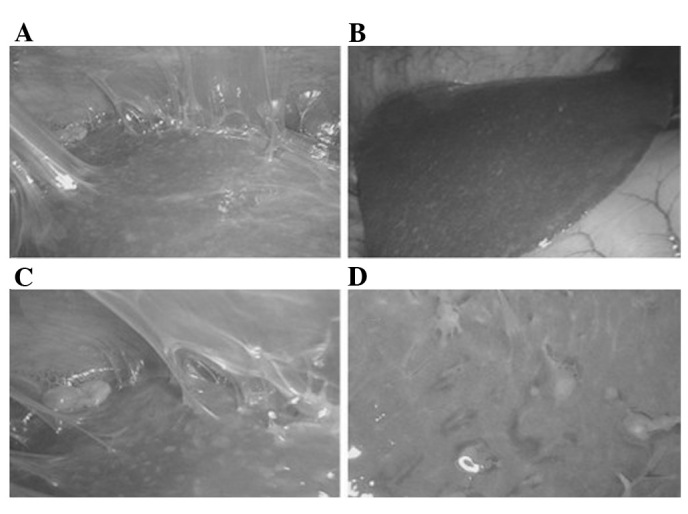
Laparoscopy results of the patient after treatment. (A) Tuberculous peritonitis symptoms of the right lobe of the liver and (B) left lobe were impoved. (C) Left lobe adhesion of liver presented fibrin network. (D) White nodules were greatly reduced.

After antituberculous drug treatment was conducted, the patient left hospital on May 1, 2010, and the disease condition was stable. The patient received a second laparoscopy before termination of antituberculous drug treatment to confirm that the disease status was improved. The patient was hospitalized at the Department of Tuberculosis (The First Affiliated Hospital of Xinxiang Medical College) and the laparoscopy was conducted in early May 2011. At this time, macroscopic examination results were improved (Fig. 4). Tuberculous peritonitis symptoms of the right lobe of the liver (Fig. 4A) and the left lobe of the liver (Fig. 4B) were impoved. White nodules were greatly reduced (Fig. 4D). In addition, yellow-white nodules on the liver surface were greatly reduced, and the extent of whitening of liver capsules was also improved. However, the left lobe adhesions of the liver presented a fibrin network (Fig. 4C). Numerous fibrin networks adhering to the peritoneum (typical symptoms of tuberculous peritonitis) caused by the early inflammation were still present.

Table I shows the clinical progress of CRP level normalization after the patient was treated with antituberculosis drugs INH, RFP, PZA and SM. One week after the second laparoscopy, the patient was released from the hospital, and the antituberculous drug treatment was terminated. According to the data of this case, we suggest that laparoscopy may be used to examine the development of tuberculous peritonitis.

## Discussion

Tuberculous peritonitis may be diagnosed by directly coating the ascites sample onto a slide to conduct Gram staining or culture *Mycobacterium tuberculosis* from the ascites sample. However, these methods are usually unreliable (3). The sensitivity of Gram staining ranges from 0 to 6% and the majority of staining results of tuberculous ascites are negative (4). Laparoscopy and biopsy are believed to be extremely useful for the identification and diagnosis of tuberculous peritonitis, cancer or infectious disease (5). Compared with the diagnosis rate of CT imaging examination (69%), the definitive diagnosis rate of a combination of three laparoscope examinations and a biopsy of unknown-source ascites reaches 85–100%.

Another advantage of laparoscopy is that its result is obtained far earlier than that of the traditional microbiology approach, such as ascites culture or PCR method, which usually require 2–4 weeks. For patients with tuberculous peritonitis, the failure rate of laparoscopy may reach 16%, and is not completely risk-free (6). Laparoscopy is able to reveal the typical fibronectin forms of tuberculous peritonitis, called a fibrin network. Intestinal perforation is a serious complication and currently it is believed that intestinal perforation usually appears in fibronectin-type tuberculous peritonitis. In this study, the first laparoscopy revealed no peritoneal adhesion, while the second laparoscopy showed fibrin networks with obvious adhesion. This phenomenon suggests that early laparoscopy examination was safer than the late stage for tuberculous peritonitis.

The characteristics evident with tuberculous peritonitis laparoscopy are the presence of multiple yellow-white nodules and parietal peritoneum ascites on the visceral surface. It is extremely difficult to differentiate these nodules from hepatic sarcoidosis. In this study, this type of nodule on the liver surface was present, and direct Gram staining of the nodule sample showed *Mycobacterium tuberculosis* infection. In addition, hepatic biopsy results demonstrated the presence of epithelioid cell granuloma. These results confirmed the diagnosis of tuberculous peritonitis. Laparoscopy and biopsy contribute to the rapid and definitive diagnosis of tuberculous peritonitis. Peritoneal or visceral adhesion and occasional peritoneal inflammation and bleeding are possible symptoms of tuberculous peritonitis. After the specific antituberculous drug treatment, the second laparoscopy of the patient showed that the yellow-white nodules on the liver surface disappeared. However, numerous fibrin networks adhering to the peritoneum were still noted, which is the typical symptom of tuberculous peritonitis and the result of early inflammation. When the patient was nearly cured, the laparoscopy results revealed that the yellow-white nodules on the liver surface were greatly reduced. Therefore, laparoscopy may be used for the evaluation of treatment efficiency. However, it is necessary to consider the operation risk caused by *Mycobacterium tuberculosis*-induced adhesion in the case of subsequent laparoscopy.

## Figures and Tables

**Table I:** Comparison of reaction time between the intervention and surgical treatment CRP (mg/l).

	Time (months)							
	1	2	3	4	5	6	12	16
CRP mg/l	75	16	12	11	11	10	9	5

CRP, C-reactive protein.
